# Advances in Phenotyping Obesity and in Its Dietary and Pharmacological Treatment: A Narrative Review

**DOI:** 10.3389/fnut.2022.804719

**Published:** 2022-02-15

**Authors:** Roberta Pujia, Maria Grazia Tarsitano, Franco Arturi, Antonino De Lorenzo, Andrea Lenzi, Arturo Pujia, Tiziana Montalcini

**Affiliations:** ^1^Department of Medical and Surgical Science Nutrition Unit, University Magna Grecia, Catanzaro, Italy; ^2^Department of Biomedicine and Prevention, University of Tor Vergata, Rome, Italy; ^3^Department of Experimental Medicine, University La Sapienza, Rome, Italy; ^4^Department of Clinical and Experimental Medicine, Nutrition Unit, University Magna Grecia, Catanzaro, Italy

**Keywords:** obesity, obesity phenotypes, sarcopenia, body composition, fat mass, muscle mass, dietary treatment

## Abstract

In recent times, it has become evident that there are individuals who, from a metabolic point of view, are affected by obesity but have a normal body mass index. There are also metabolically healthy individuals with a high body mass index who are thus are considered as to be affected by obesity obese. Understanding that individuals with obesity are phenotypically heterogeneous is a relatively novel concept which, although present in the scientific literature, unfortunately has not yet had an impact in clinical practice. However, common dietary approaches are not effective in treating large numbers of obese patients with obesity. This narrative review, based on the material searched *via* PubMed and the Web of Science up to October 2021, proposes a downsizing of the role of the body mass index in identifying the individual with “true obesity” since it is only partially useful, and suggests a new approach which also integrates the body composition and assessment of metabolic parameters. This approach leads to personalized therapies that work best for each obesity phenotype in reducing the risk of non-communicable diseases.

## Introduction

Worldwide obesity has nearly tripled over the past 50 years (1). In this period, the prevalence of obesity, and especially severe obesity, has increased in adults by 42% (2), thus currently over 650 million adults are affected by obesity, while more than 1.9 billion adults are overweight (1). By 2030, the respective number of adults who are overweight and affected by obesity is projected to be 2.16 billion and 1.12 billion, respectively (3).

The global deaths and disability-adjusted life years (DALYs) attributable to a high body mass index (BMI) were analyzed in the Global Burden of Disease (GBD) 2017 study (4). Cardiovascular disease (CVD) was found to be the leading cause of high BMI-related DALYs, followed by diabetes, kidney disease, and neoplasms. These conditions together accounted for 89.3% of all high-BMI-related DALYs (4). The GBD 2017 study demonstrated that a poor diet is responsible for more deaths than any other risk factor in the world, leading to one in five deaths being linked to unhealthy diets (4, 5). Obesity is thus an urgent problem that needs to be properly addressed. Physicians, public and global health policy decision-makers need timely, up-to-date scientific information to develop new interventions aimed at counteracting the burden generated by obesity. The importance of this has been magnified by the current COVID-19 pandemic, as obesity and the related chronic diseases are among the strongest predictors of COVID-19 severity and mortality (6).

Interest in the association between body shape and health outcomes date back over a century (7–9). However, it is now well recognized that more efficacious anthropometric biomarkers are required to better predict the onset of non-communicable diseases (NCDs) than BMI as well as new, well-functioning and tailored dietary/nutritional interventions for individuals affected by obesity (7, 8).

The benefits of this new approach are supported by three factors. First, we now have several advanced technologies that can identify the changes in body composition that precede the onset of NCDs. Second, it is now known that individuals with obesity are only apparently similar, whereas in fact they are phenotypically (and genotypically) heterogeneous. Third, no single management strategy is suitable for each individual patient and novel therapeutic approaches for treating particular obesity phenotypes and tailoring the treatment are already being generated in response to the new biological drivers.

This narrative review is based on the material searched for and obtained via PubMed and the Web of Science up to October 2021. The search terms used were: “obesity, obesity phenotypes, obesity paradox, adipose tissue, fat mass, lean mass, muscle mass, sarcopenia, DXA, body composition” in combination with “non-communicable disease, chronic diseases, cardiovascular, coronary heart disease, mortality, dietary pattern.”

## Anthropometric Parameters as Biomarkers of Chronic Disease

The BMI, which is calculated by a person's weight in kilograms divided by the square of his or her height in meters (kg/m^2^), was initially used as a good indicator of obesity and correlated well with the development of several NCDs such as type 2 diabetes (T2DM), several types of cancer, osteoarthritis, asthma, and all-cause mortality (10–16). The BMI is widely used primarily because it is a simple, non-invasive and inexpensive test, which can be used at the population level to generate models that span geographical regions.

However, the BMI has several limitations. In fact, it cannot be used as a proxy of body fat content in individuals who tend to have a high lean body mass (LBM) (17). One meta-analysis suggested the protective effect of a high BMI on mortality and found that only severe obesity increased the risk of CVDs (18), thus, highlighting the BMI's limitation in clarifying the diverse body compartments (fat mass-FM, muscle mass-MM, etc.). In addition, in the Münster Heart Study (PROCAM), the risk of coronary heart disease (CHD) was mediated via other risk factors (19). All this evidence has generated uncertainty regarding the risks associated with obesity.

Are waist circumference (WC) or the waist-to-hip ratio (WHR) better biomarkers than the BMI? In 1947, the French physician Vague observed that his patients with both obesity and diabetes or clinical signs of a CVD had a central distribution of body fat and that the gynoid fat accumulation was rarely associated with these complications (20). However, very recently the medical community has recognized that WC and WHR are more strongly correlated to metabolic complications and cardiovascular outcomes than the BMI (21–23).

Other measures of body fat, and particularly visceral fat, seemed to be better indicators of the risk of obesity-related health issues (24, 25). In the INTERHEART study, the WHR was the strongest anthropometric predictor of myocardial infarction in both genders, across all age and ethnic groups, in smokers and non-smokers, and in those with or without the classical CV risk factors that are the consequences of obesity (26). A meta-analysis reported the superiority of centralized obesity measures, especially the waist-to-height ratio (WHtR) over the BMI for CVD risk detection (27). The association of the conventional BMI parameter with myocardial infarction was found to be weaker and less consistent across ethnic and other subgroups (26). In addition, neck circumference (NC) has been proposed as a quicker, more reliable and easier-to-apply anthropometric marker of central obesity (28). NC also predicts cardio-metabolic risk factors (28).

Results from the EPIC (European Prospective Investigation into Cancer and Nutrition) highlighted the use of the central obesity index in predicting the risk of death (29). In addition, the scientific literature continues to confirm the association between abdominal obesity and the risk of cancer (30–32). However, there could be considerable differences in the percentage of fat and LBM or MM between individuals with similar WC, WHR, NC, or BMI especially when these indices are compared across different ethnic groups (33).

In general, none of these anthropometric parameters differentiate between FM and MM, which have opposite health impacts. The main drawback of WC (and WHR) is also an inability to differentiate subcutaneous from visceral fat deposition. Even with the same WC value, a larger subcutaneous adipose tissue is observed in the gynoid region of an older female compared to a younger female (34). WHR and WC are, thus, not accurate indicators of abdominal visceral fat accumulation (35). Furthermore, WHR and WC measurements need standardization and training as there are several methods described for assessing these parameters using non-elastic flexible tape.

## Novel Nutritional Biomarkers of Non-communicable Diseases

Biomarkers play an important role in the evaluation of the onset of chronic diseases as well as in the development of drug treatments for these conditions (36). Biomarkers may also be able to reflect the pathophysiological process of a specific disease and may be able to predict the prognosis and guide clinical decision making (37). Accuracy, precision, high sensitivity and specificity and low intra-individual variability are important characteristics of an ideal biomarker (36).

Because of the great advantages offered by imaging tools in research and clinics, the focus of clinicians is now moving to powerful imaging techniques such as computed tomography (CT), magnetic resonance (MR) imaging, dual-energy X-ray absorptiometry (DXA), or other techniques such as bioimpedance analysis (BIA) and ultrasound (US) to more accurately measure FM (38). Although some of the above methods can be expensive, in some cases invasive, and not readily accessible, they are more accurate at measuring body fat, and thus better at predicting the risk of obesity-related health issues. One limitation of using these tools is the need for specialized equipment and trained staff, which can be challenging in routine clinical practice.

In two large prospective cohort studies, a strong and linear association was found between FM, assessed by radiological imaging techniques, and mortality from all causes (39, 40), CVD, and cancer (40). In a cohort of postmenopausal women from the Women's Health Initiative (WHI) cohort, DXA-derived FM measures were positively associated with breast cancer risk (41). Among postmenopausal women with a normal BMI, both elevated trunk fat and reduced leg fat, assessed by DXA, were associated with an increased risk of CVD (42). However, FM should be normalized for body size, precisely to eliminate the differences in the %FM associated with one's height (43). The fat mass index (FMI), which is calculated by dividing FM by the square of height, could be therefore, a useful measure of obesity, better than FM alone.

However, the relationship between the risk of FM and NCDs is also influenced by the presence of sarcopenia, a clinical condition in which low muscle function is associated with a low quantity or quality of MM (44). In fact, in a large prospective cohort study, free-fat mass (FFM), rather than FM, was a stronger predictor of overall cancer risk (45). The discovery of the role of LBM in the mortality risk in patients with cardiac disease (46, 47), suggested that other body composition parameters, such as MM and appendicular skeletal muscle mass (ASMM), could be more appropriate biomarkers of NCDs. The prevalence of sarcopenia is significantly higher in individuals with different type of NCDs, with the highest prevalence in individuals affected by T2DM, CVD, dementia, and respiratory disease (48).

There is a particular phenotype of individual in whom, despite an excess of FM and a high BMI, the CV risk is low or not increased due to a preserved or high MM. Some authors have defined this phenomenon as “the obesity paradox,” in which obesity seemed to protect against CV diseases (47, 49, 50). The study of body composition and MM has clarified that obesity is not protective against NCDs unlike that maintaining MM and, thus there is a “BMI paradox,” rather than an “obesity paradox” (50). On the other hand, sarcopenia could worsen the effects of obesity, especially in older adults, resulting in a particular phenotype defined as “sarcopenic obesity” which was found to have a higher risk of all-cause mortality than obesity or sarcopenia alone (51).

Overall, all these studies highlight that, although clinically valuable, classical, old anthropometric measures have a limited sensitivity and specificity as screening modalities and are poorly predictive of clinical outcome. Understanding that individuals with obesity are phenotypically heterogeneous is a relatively novel concept which, although present in the scientific literature, unfortunately has not yet had an impact in clinical practice.

## Progress in the Identification of Different Obesity Phenotypes

On the basis of the current scientific data, it is clear that individuals with obesity are a heterogenous group, probably requiring specific treatments.

It has become evident that there are individuals who are affected by obesity but have a BMI in the normal range, as well as metabolically healthy individuals with a high BMI, thus considered with obesity. We propose a downsizing of the role of the BMI in identifying individuals with “true obesity” which should be used if integrated with body composition and metabolic assessment.

A screening strategy in phenotyping individuals with obesity is thus urgently needed in the global strategy aimed at preventing NCDs. Image diagnostics, supported by technological innovations, provides an accurate and repeatable body composition assessment. Among the number of available technologies, bioimpedance analysis (BIA) and DXA could play a primary role as they are minimally or non-invasive, cost effective, easy to perform and, of course, accurate, as previously described (38).

**Figure 1:**
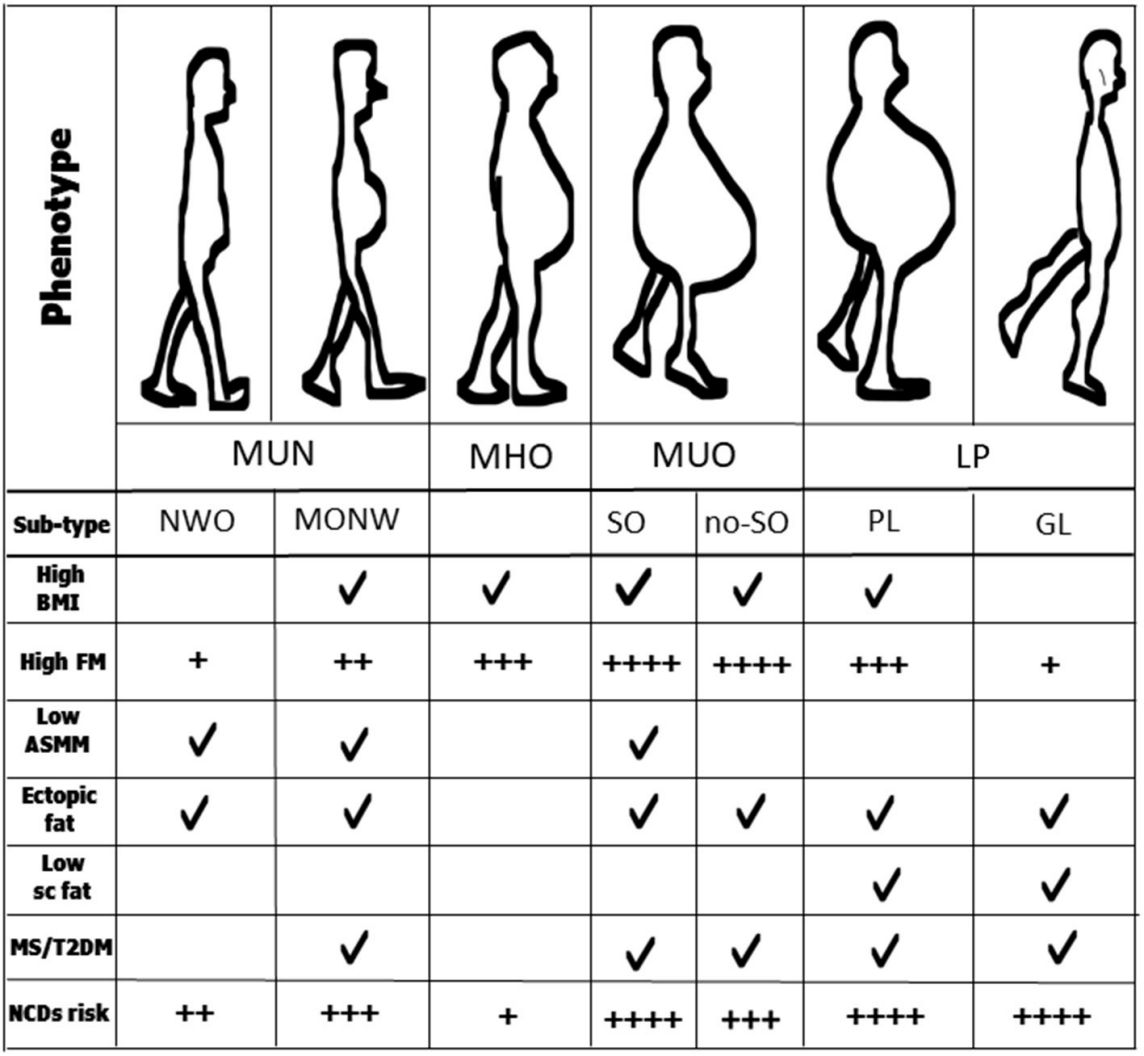
Anthropometrics and other clinical characteristics of obesity phenotypes and sub-types. MUN, normal-weight obesity; MHO, metabolically healthy obesity; MUO, metabolically unhealthy obesity; LP, lipodystrophic phenotype; NOW, normal-weight obesity; MONW, metabolic obesity normal-weight; SO, sarcopenic obesity; no-SO, no sarcopenic obesity; GL, generalized lipodystrophy; PL, partial lipodystrophy; BMI, body mass index; FM, fat mass; ASMM, appendicular skeletal muscle mass; MS, metabolic syndrome; T2DM, type 2 diabetes; NCDs, non-communicable diseases.

Here we thus suggest that, on the basis on body composition assessment and metabolic status, and only in part on BMI, individuals with “true obesity” could be better identified. The term “obesity” therefore assumes a new and different connotation with respect to the meaning assumed up to now. Specifically, based on the integration of all these parameters, people could be categorized as having:

1. metabolically healthy normal-weight (MHN) (healthy individuals);
2. metabolically unhealthy non-obesity [MUN, which includes two different sub-phenotypes, previously identified as normal-weight obesity, NWO (46) and metabolic obesity normal-weight, MONW (52)];
3. metabolically healthy obesity (MHO);
4. metabolically unhealthy obesity [(MUO) (53), also defined as “complicated obesity” and which includes sarcopenic obesity-SO (54)];
5. lipodystrophic phenotype (LP), (Figure 1).

Based on BMI, only MHO and MUO would be defined as having obesity, while based on body composition assessment and metabolic characteristics, MUN (which include overweight individuals or those with a cluster of metabolic features as dyslipidemia and hyperglycemia) and LP (who have an abnormal fat accumulation) are also considered individuals with “true obesity.”

### Epidemiology and Risk of Chronic Diseases Among Obesity Phenotypes

The real prevalence of these phenotypes around the world is not entirely known. The prevalence of MHO is estimated overall to be 6.5-10%, while it is about 30% in individuals with obesity. The prevalence of MUN is 20-30% in the general population with the NWO prevalence ranging from 4.5 to 23.5% and MONW from 10 to 37% among individuals with a BMI of <25 kg/m^2^ (44, 55, 56). LPs occur very infrequently, and thus are considered rare diseases.

Do these phenotypes have a different risk in terms of chronic diseases? Overall studies suggest a different degree in the risk of NCDs between the various obesity phenotypes, thus individuals with MUO are at higher risk than those with MUN, and individuals with MUN are at higher risk than those with MHO (53, 55, 57–59).

The cardiometabolic risk associated with MHO is still an open issue (60). A recent population-based prospective cohort study of 381,363 UK Biobank participants with a median follow-up of 11.2 years demonstrated that people with MUN, MHO and MUO were at a substantially higher risk of diabetes, atherosclerotic cardiovascular diseases (ASCVD), heart failure (HF), respiratory diseases and all-cause mortality compared with people with MHN (53). It is worth noting that people with MHO were at an even higher risk of HF and respiratory disease than those with MHN and MUN (53, 55). A multi-national European study found that those with MHO had a higher CVD risk than those with MHN but lower than those with MUN and MUO (56). Among people with baseline MHO who remained affected by obesity, over one-third became metabolically unhealthy within 3-5 years (53, 55, 61). These people acquired an even higher risk of ASCVD (53). Due to the presence of several metabolic alterations, including hyperglycemia and insulin resistance, high blood pressure and hyperlipidemia (46, 59, 62), people with MUN also have a high risk of NCD (53).

Major causes of mortality for LP include heart disease (particularly heart failure and myocardial infarction), liver and kidney failure, and acute pancreatitis (63).

### How to Differentiate Between the Phenotypes?

Recent studies highlight an important phenotypical difference between individuals with obesity. While individuals with MHO seem to have higher physical activity levels than those with MUO (64, 65), individuals with MUN (both NWO and MONW) have a poorer MM, in terms of quality and quantity than MHO and MHN (44, 56, 65–68). Furthermore, since they do not manifest the metabolic syndrome (MS) despite a cluster of metabolic and genetic features, individuals with NWO differ from those with MONW, who in contrast, might have MS (69). In addition, individuals with NWO have a normal BMI, while those with MONW may be overweight (69, 70). However, an important common feature is that both are at risk of sarcopenia (46, 70). Figure 1 presents all these characteristics.

Lipodystrophies constitute a rare group of heterogenous genetic or acquired disorders, which are mainly characterized by partial or total loss of adipose tissue, especially in the subcutaneous adipose depots, of individuals with a wide range of BMIs and, as a result of the inability to store energy, with ectopic fat accumulation (63). Limited lipid storage capacity in subcutaneous fat depots results in the near total lack of adipocyte expandability in patients with generalized lipodystrophy, in which subcutaneous fat is absent on the face, arms, legs and buttocks and who also express acromegaloid features (63, 71). In individuals affected by partial lipodystrophy, the inability to store energy in the subcutaneous depots is partial and leads to an increased WHR due to a low ratio of the lower limb to truncal fat or in contrast, in certain forms, an increased subcutaneous fat deposition in the lower extremities (63, 71).

### Metabolic Characteristics of the Different Obesity Phenotypes

It has been now recognized that excessive fat mass alone does not increase the risk of T2DM. Various recent studies have shown a link between a low MM, especially appendicular muscle mass (ASMM), and the development of T2DM (72–74), which may partially explain the high risk of CVD and cancer in NWO and MONW phenotypes.

Central adiposity is one of the principal characteristics of MUO, which provides the foundation for the increase in the flow of free fatty acids (FFAs) and the inhibition of insulin release. The large number of FFAs contributes to reducing the glucose up-take by skeletal muscle and stimulates the hepatic production of very low-density lipoproteins (VLDLs) and glucose. FFAs also have a lipotoxic effect in the pancreatic beta cells, leading to the development of T2DM.

In MUO, the risk of T2DM rises more than 10-fold compared to healthy individuals and the CV risk is twice in comparison with MHN (53, 75). In MUO associated with multiple risk factors, the risk of developing the disease is greater than the sum of the risks attributable to each individual factor (73). The association between visceral fat and metabolic and cardiovascular disorders is also related to the accumulation of ectopic fat that accompanies visceral adiposity (76, 77). Furthermore, insulin and inflammation are the main actors in the pathogenesis of muscle loss in those with obesity (78), which mainly occurs in the early stages of the aging process (54). Therefore, in this review, SO is considered as a subtype of the MUO.

If obesity is defined as abnormal fat accumulation that presents a risk to health, individuals with lipodystrophy have a particular obesity phenotype. Individuals with LP are also affected by insulin resistance, severe hypertriglyceridemia, diabetes, and liver steatosis (63, 71, 79, 80). Figure 1 summarizes all these metabolic alterations.

## Dietary, Physical Exercise and Pharmacological Treatments Tailored to the Different Obesity Phenotypes

Although it is well accepted that the BMI does not capture the large heterogeneity in the risk of NCDs observed across individuals, only a few studies have tested the effects of lifestyle interventions in relation to the different obesity phenotypes.

### Dietary, Physical Exercise, and Pharmacological Treatments in MUN

It is intuitive that a significant weight loss may only be partially practical, or not practical at all, for individuals with MUN. Individuals with MUN (both NWO and MONW) have less lean mass (44, 56, 65–68) than other phenotypes. One study demonstrated that individuals with NWO have similar diet quality scores but lower physical fitness levels than lean individuals and, thus, a higher quality of diet than those who are overweight or suffer from obesity (81). Physical exercise prevents the development of an NWO phenotype (82). A program of resistance exercise combined with a dairy supplement significantly decreases FM and improves the metabolic parameters better than a standard supplement in overweight individuals with low MM (83). A 6-month soy-enriched high protein snack meal in women with NWO increases ASMM and reduces FM and appetite compared to an isocaloric low protein snack (84). Twelve weeks of a high protein diet results in no significant weight loss in women with NWO, but in a significant improvement in LBM and a reduction in FM with respect to a standard protein diet (85). Taken together these studies suggest that, in terms of body composition and metabolic parameters, physical exercise and improvement in MM might be more important than a dietary approach in MUN.

**Figure 2:**
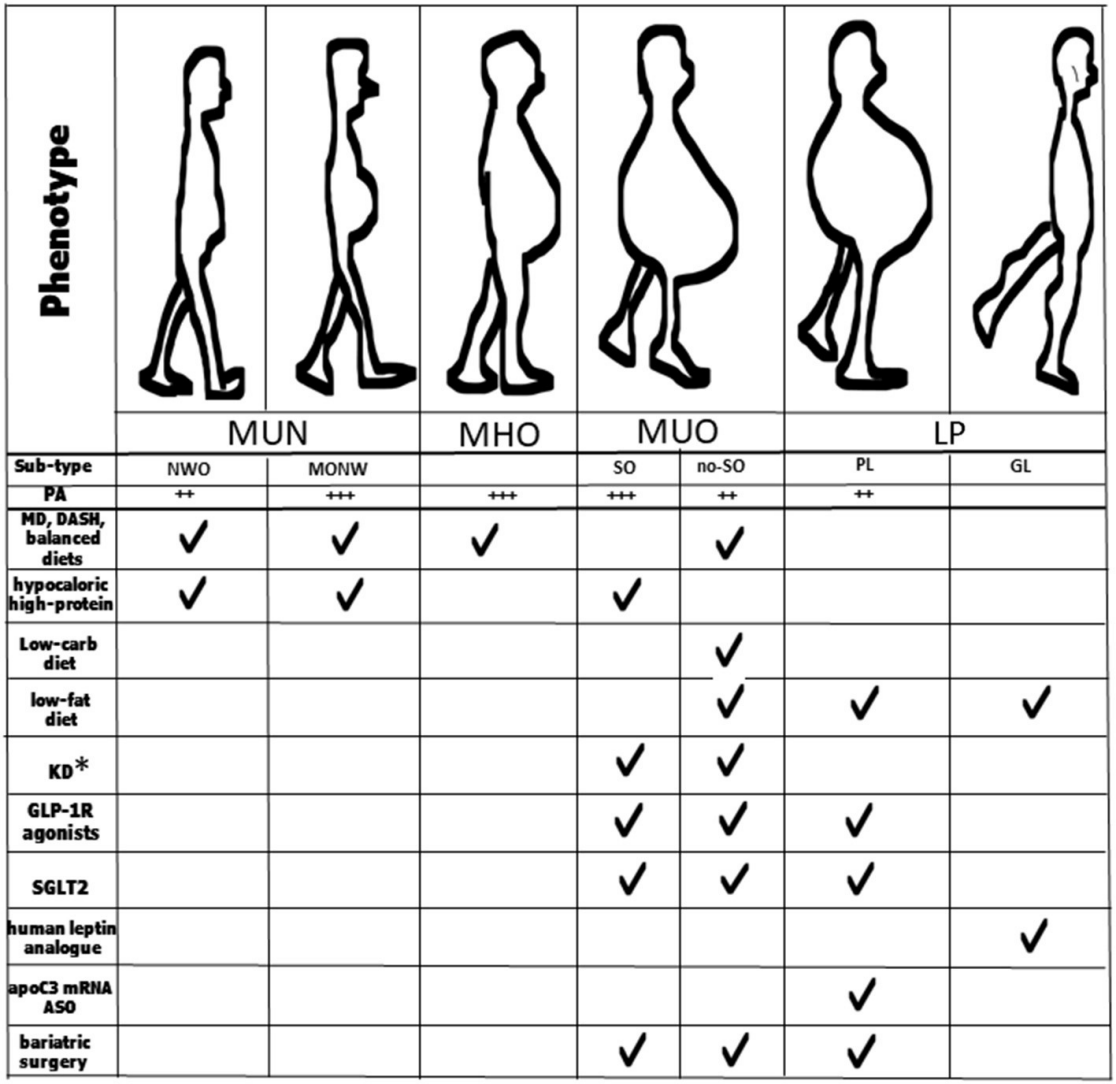
Key strategies for management of the different obesity phenotypes and sub-types. *Patients taking SGLT2 inhibitors should avoid KD; MUN, normal-weight obesity; MHO, metabolically healthy obesity; MUO, metabolically unhealthy obesity; LP, lipodystrophic phenotype; NOW, normal-weight obesity; MONW, metabolic obesity normal-weight; SO, sarcopenic obesity; no-SO, no sarcopenic obesity; GL, generalized lipodystrophy; PL, partial lipodystrophy; MD, Mediterranean diet; DASH, Dietary Approaches to Stop Hypertension; KD, ketogenic diet; GLP-1 R, glucagon-like peptide-1 receptor; ASO, antisense therapeutic oligonucleotide.

In the MONW phenotype, a high quality diet score, linked to a high consumption of fruit and vegetables, is significantly associated with a reduction in the risk of all-cause mortality (22%; HR, 0.78; 95% CI, 0.68-0.90) and CV mortality (HR 0.79; 95% CI, 0.65-0.97) (86), especially in young adults (87). Healthy dietary patterns alone may thus be effective in reducing the cardiometabolic risk among younger adults with MONW, but in older adults, physical activity might be more important than a dietary approach (88) (Figure 2).

### Dietary, Physical Exercise, and Pharmacological Treatment in MHO

As discussed above, MHO is a transient phenotype and over one-third of individuals become metabolically unhealthy overtime (53, 58, 89). MHO individuals with an impaired fat oxidation rapidly develop MS/diabetes (90), gain body weight, and suffer co-morbidities (91–94). Skeletal muscle contributes significantly to the overall utilization of fat (95), and after several sessions of vigorous intensity exercise (96, 97) or whole-body electromyostimulation (WB-EMS), fat oxidation significantly improves (98). In MHO, exercise training is necessary to improve fat oxidation (99). Furthermore, several studies have demonstrated that certain dietary bioactive components (such as catechins, capsaicin, and L-carnitine) significantly improve fat oxidation, with a consequent reduction in BMI and FM (100–102).

In one study, following a Mediterranean diet (MD), which is rich in antioxidants, was positively associated with the MHO phenotype, but not in the older age group (87). A five-point increase in the adherence to MD in MHO individuals was associated with a 41% reduction in the risk of all-cause mortality (HR, 0.59; 95% CI, 0.37-0.94) (86) (Figure 2). All these studies seem to indicate that it might be possible to prevent the transition from MHO to MUO through intense physical activity programs as well as through the consumption of foods that contain bioactive molecules that stimulate fat utilization as in the MD, or through nutraceuticals.

### Dietary, Physical Exercise, and Pharmacological Treatment in MUO

Although specific features of a diet, such as a low glycemic index, might slightly lower the risks of CVD (103), a dietary approach alone (even an MD), does not reduce mortality in the MUO phenotype (86). A limited body weight reduction (5-20%) has several beneficial metabolic effects on serum glucose and LDL and blood pressure (104), and thus on CVD risk.

A recent meta-analysis, based on quantitative estimates of the relative effect of different diets, demonstrated that both MD and DASH result in slightly less weight loss (~3 vs. ~5 kg, at 6 months) and blood pressure reductions (~3 vs. ~ 6 mmHg) than low-carbohydrate or low-fat dietary patterns (105, 106) (Figure 2). A diet relatively low in carbohydrates may have positive effects on muscle protein turnover and prevent glucose abnormalities, especially in the elderly (107). One variation of a carbohydrate restriction diet is the ketogenic diet (KD) which consists of up to 70% of fat and below 50 g of carbohydrate of the total daily calorie intake. There is evidence that the use of the KD in MUO patients improves insulin sensitivity and glycemic control and is successfully used as part of the treatment for T2DM, obesity and metabolic syndrome (108–110) (Figure 2). From a weight loss perspective and in the short term, a low-carbohydrate diet thus appears to be more effective than other diets in MUO.

However, low-carbohydrate diets result in reduced effects compared to low-fat diets, MD and DASH on LDL cholesterol reduction (105). On the other hand, in several studies LDL-C was found to slightly increase as a consequence of a low-carbohydrate diet (105, 106). Two cohort studies seem to deny any maintainable benefit from a low-carbohydrate diet in the general population (111). Randomized clinical trials and prospective cohorts studies have demonstrated that carbohydrate quality may be relevant for health in the long-term, and a high consumption of whole carbohydrate foods have been shown to reduce NCD incidence (112–114). A meta-analysis demonstrated that there is no difference in changes in HbA1c between low-carbohydrates and balanced weight loss diets at 3–6 months and 1–2 years (115), and glycaemia depends on the degree of weight loss in overweight adults and those with obesity rather than the type of diet (104). A prospective cohort study using data from the US National Health and Nutrition Examination Survey (NHANES) from 1999 to 2014, suggested that total mortality depends on the quality and food sources of macronutrients (116) as already suggested from studies on the glycemic index (103).

Taking together, these studies suggest that, in order to obtain rapid effects on body weight loss and metabolic parameters, a low-carbohydrate diet is useful for a short period, and then patients should switch to a balanced and high carbohydrate (whole grains, fiber) long-term diet to prevent NCDs.

However, most individuals with severe obesity, i.e., those with MUO, often fail to maintain long-term weight loss with the associated metabolic improvement (117), thus pharmacological agents are needed for these individuals. Newer pharmacological treatments have shown promising results in terms of weight loss and the prevention of obesity-related CV complications in MUO.

Liraglutide, a GLP-1(glucagon-like peptide-1) receptor agonist induces and maintains weight loss by promoting satiety and reducing energy intake in patients with obesity affected by T2DM, hypertension and dyslipidemia (Figure 2). Liraglutide is thus now approved for treating obesity at an increased 3.0 mg daily dose (118–120). In a large, multicenter, double blind trial carried out in patients with T2DM and high cardiovascular risk, at a daily dose of 1.8 mg, liraglutide significantly reduced the primary composite outcome, which included death from cardiovascular causes, non-fatal myocardial infarction, or non-fatal stroke, compared to a placebo over 3.8 years (13.0 vs. 14.9%; HR 0.87, 95% CI 0.78-0.97), with fewer CV deaths in the treated group compared with the placebo (hazard ratio, 0.78; 95% CI, 0.66-0.93) (121).

Semaglutide is an oral GLP-1 receptor agonist approved for the treatment of T2DM which reduces HbA1c by ~0.9%, body weight by ~3.0 kg, systolic blood pressure by 3.2 mmHg as well as all-cause and cardiovascular mortality compared with placebo (122). However, it is associated with an increased incidence of adverse gastrointestinal events (122).

SGLT2 inhibitors are a new group of oral medications for treating T2DM which have demonstrated efficacy for weight loss and the reduction of cardiovascular risk in individuals with obesity (123, 124). However, due to the risk of euglycemic diabetic ketoacidosis in the setting of SGLT2 inhibitor use and KD, patients taking SGLT2 inhibitors should be advised to avoid this type of diet (Figure 2).

Fixed-dose combination of Naltrexone-Bupropion became available in 2014 after FDA approval for use as an adjunct to a reduced-calorie diet and increased physical activity in long-term weight management in individuals with obesity (125). Bupropion inhibits the presynaptic reuptake of both dopamine and noradrenaline (key neurotransmitters in the reward pathway associated with addiction) leading to increased levels of both these neurotransmitters in the synaptic cleft. Naltrexone belongs to a group of drugs known as opioid antagonists and regulates satiety. In several studies, Naltrexone-Bupropion demonstrated a placebo-adjusted weight loss of 2.5-5.2% at target doses (104). However, the FDA initially rejected this new drug application for Bupropion because of the rise in blood pressure and heart rate. The cardiovascular safety of this combination remains uncertain (126).

Metabolic/bariatric surgery is the most effective strategy to accomplish a significant (≥30%) and durable (at ≥5 years) weight loss in patients with obesity (104) and is recommended for patients with a BMI ≥ 40 kg/m^2^ or ≥ 35 kg/m^2^ with obesity-related comorbidities.

In addition, among patients with T2DM and a BMI of 30 or greater, compared with usual care, metabolic surgery (which included Roux-en-Y gastric bypass, sleeve gastrectomy, adjustable gastric banding, and duodenal switch) was associated with a significantly lower risk of major adverse cardiac events (MACE) and diabetic nephropathy (127). On the other hand, there is only a transient positive effect of abdominal lipectomy in reducing FM and body weight in women, which disappears a few months after the procedure (128, 129).

Regarding sarcopenic obesity, this syndrome is predominantly observed in the aging population which is thus at risk of several complications from both sarcopenia and obesity (130). The most effective lifestyle intervention for treating sarcopenic obesity should include both diet-induced weight loss and regular exercise (aerobic and resistance exercises) (131, 132). In older adults with both obesity and sarcopenia, a 20% reduction in body weight results in a greater reduction in FM than LBM, leading to an increase in MM (133). A hypocaloric high-protein diet (1.2-1.4 g/kg body weight reference/day) or KD preserve LBM compared to a low-calorie diet (134). An ingestion of at least 25 grams of protein per meal ensures an optimal muscle protein synthesis, especially in the elderly (135) (Figure 2).

### Dietary and Pharmacological Treatment in LP

There are few studies on specific diets in lipodystrophy. Diets high in fat should be avoided in patients with LP due to the possible development of metabolic sequelae while an energy-restricted diet and the consumption of medium-chain triglyceride oil formulas can improve metabolic abnormalities in these individuals (71). Metreleptin, a synthetic analog of human leptin that binds to and activates the leptin receptor, was first approved by the U.S. Food and Drug Administration (FDA) in 2014 for the treatment of metabolic complications in patients with congenital or acquired generalized lipodystrophies (136). Metreleptin was then approved by the European Medicines Agency (EMA) for adults and children aged ≥ 12 years affected by partial lipodystrophy who are non-responders to standard treatments (137). However, metreleptin is also effective in treating partial lipodystrophies (138). Volanesorsen is a second-generation chimeric antisense therapeutic oligonucleotide (ASO) which selectively reduces apoC3 mRNA, thereby lowering the levels of triglycerides and FM in particular genetic syndromes such as familial partial lipodystrophy (139, 140). It was approved in the European Union in May 2019 (Figure 2).

## Implications for Clinical Practice and End Social Stigma

Current knowledge and technologies enable detailed assessments of the body composition of individuals so that their treatments can be tailored. Although these technologies have come down in cost, their availability is limited, especially in peripheral hospitals. Moreover, there is often a scarcity of skilled personnel in this area, which thus preclude phenotyping obesity in routine clinical practice. Getting a DXA in some countries is really challenging and the cost of the scan may be prohibitive (141, 142).

BIA and DXA are currently mostly used in research, rather than in general hospitals (141), and sarcopenia is still a relatively new concept (141). Governments seem more likely to carry on with the old strategies and policies for patients with obesity rather than implementing innovative policies. Consequently, some of the possible approaches outlined in this review may take time to be accepted by decision makers in public health and the impact of this review in clinical practice is thus difficult to predict.

According to the WHO (143), weight bias is defined as a negative attitude toward, and belief about, others because of their weight. Obesity stigma is a result of weight bias and an individual with high body weight is victim of prejudice. Obesity stigma can affect an individual's everyday life. Increasing academic and public education regarding the complex causes of obesity and its phenotypes can help end obesity stigma. This narrative review highlights that the diagnosis of obesity is complex and goes beyond body weight. Even a lean individual could be metabolically obese. It is therefore better not to judge other individuals (or patients) by weight.

## Open Questions

In the elderly, a combination of aerobic and resistance exercises appears to be the most effective treatment strategy in helping to decrease body weight and improve MM function and fitness. However, how we can really make a positive impact on their daily behavior is not yet clear.

Further research is also needed to understand the magnitude of the reduction in the burden of NCDs by addressing the different phenotypes of obesity.

## Conclusions

Obesity is a clinical condition associated with metabolic derangements resulting in severe comorbidities as well as the risk of the development of NCDs that depend on the phenotype and subtype of obesity. Many complications of obesity are secondary to an excess of adipose mass resulting in ectopic lipid storage in other organs and causing insulin resistance. In fact, insulin resistance leads to diabetes, hypertriglyceridemia, and non-alcoholic fatty liver disease and cardiovascular mortality. Other complications are secondary to the loss of MM, especially of ASMM, as with most endocrine diseases, as well as physical disability and even mortality.

Common dietary approaches are not effective in treating large numbers of patients affected by obesity. These individuals thus need to undergo precise phenotyping using nuclear magnetic resonance, BIA or DXA. In fact, DXA is the reference method for body composition in terms of accuracy, non-invasiveness and cost (144). This approach leads to a tailor-made prevention and treatment strategy in order to reduce their risk of NCDs.

## Author Contributions

TM and RP: conceptualization. TM, AP, AL, and AD: supervision and review. RP, MT, and FA: original draft preparation and editing. All authors contributed to the article and approved the submitted version.

## Conflict of Interest

The authors declare that the research was conducted in the absence of any commercial or financial relationships that could be construed as a potential conflict of interest.

## Publisher's Note

All claims expressed in this article are solely those of the authors and do not necessarily represent those of their affiliated organizations, or those of the publisher, the editors and the reviewers. Any product that may be evaluated in this article, or claim that may be made by its manufacturer, is not guaranteed or endorsed by the publisher.
